# NPR3 protects cardiomyocytes from apoptosis through inhibition of cytosolic BRCA1 and TNF-α

**DOI:** 10.1080/15384101.2016.1148843

**Published:** 2016-08-05

**Authors:** Dong Lin, Yubo Chai, Reza Izadpanah, Stephen E. Braun, Eckhard Alt

**Affiliations:** a Division of Cardiovascular Diseases, Department of Internal Medicine, Mayo Clinic, Rochester, MN, USA; b Division of Clinical Pharmacology, Department of Molecular Pharmacology & Experimental Therapeutics, Mayo Clinic, Rochester, MN, USA; c School of Medicine Heart and Vascular Institute, Tulane University, New Orleans, LA, USA; d Division of Regenerative Medicine, Tulane National Primate Research Center, Tulane University, Covington, LA, USA; e School of Medicine, Heart and Vascular Institute, Tulane University, New Orleans, LA, USA

**Keywords:** apoptosis, breast cancer type 1 susceptibility protein (BRCA1), cardiomyocyte, natriuretic peptide receptor-3 (NPR3), shRNA knockdown, tumor necrosis factor α, (TNF-α)

## Abstract

Natriuretic peptide receptor 3 (NPR3) is a clearance receptor by binding and internalizing natriuretic peptides (NPs) for ultimate degradation. Patients with cardiac failure show elevated NPs. NPs are linked to poor long-term survival because of their apoptotic effects. However, the underling mechanisms have not been identified yet. Here we report the role of NPR3 in anti-apoptosis via the breast cancer type 1 susceptibility protein (BRCA1) and tumor necrosis factor α (TNF-α ). To demonstrate a role for NPR3 in apoptosis, stable H9C2 cardiomyocyte cell lines using shRNA to knockdown NPR3 were generated. The activities of caspase-3, 8, and 9 were significantly increased in NPR3 knockdown H9C2 cardiomyocytes. Knockdown of NPR3 increased the expression of BRCA1. Also NPR3 knockdown remarkably increased the activity of cAMP response element-binding protein (CREB), a positive regulatory element for BRCA1 expression. BRCA1 showed dispersed nuclear localization in non-cardiomyocytes while predominantly cytoplasmic localization in H9C2 cells. Meanwhile, NPR3 knockdown significantly increased TNF-α gene expression. These data show that NPR3 knockdown in H9C2 cells triggered both extrinsic and intrinsic apoptotic pathways. NPR3 protects cardiomyocytes from apoptosis through inhibition of cytosolic BRCA1 and TNF-α, which are regulators of apoptosis. Our studies demonstrate anti-apoptosis role of NPR3 in protecting cardiomyocytes and establish the first molecular link between NP system and programmed cell death.

## Introduction

The natriuretic peptide (NP) system includes atrial natriuretic peptide (ANP), brain natriuretic peptide (BNP), C-type natriuretic peptide (CNP) and their membrane-bound natriuretic peptide receptor 1 (NPR1), natriuretic peptide receptor 2 (NPR2) and natriuretic peptide receptor 3 (NPR3).^1,2^ NPR1 and NPR2 are membrane-bound receptors with guanylyl cyclase activity and signal by catalyzing the synthesis of the intracellular signaling molecule cGMP. NPR3 is the most widely and abundantly expressed natriuretic peptide receptor and lacks guanylyl cyclase activity. It is denoted as a clearance receptor by binding and internalizing natriuretic peptides for ultimate degradation. Furthermore, it has been demonstrated that NPR3 inhibits adenylyl cyclase activity mediated by a guanine-nucleotide-binding inhibitory (Gi) protein and down-regulates cyclic-AMP (cAMP) levels. ANP and BNP both bind to NPR1 with high affinity while CNP is the primary ligand for NPR2. All three NPs bind to NPR3 in the affinity order ANP > CNP≥ BNP.^3,4^ NPs exert their biological effects through binding to their respective receptors and are principally involved in regulating blood volume, blood pressure, ventricular hypertrophy, pulmonary hypertension, fat metabolism, and long bone growth.^5,6^

Apoptosis is tightly regulated and plays a central role in development and maintaining tissue homeostasis in all species. In mammals, apoptosis can be divided into 3 different pathways: (1) the extrinsic pathway initiated by death receptors and subsequent caspase-8 activation; (2) the intrinsic mitochondrial pathway triggered by mitochondrial release of cytochrome c followed by activation of caspase-9; and (3) the intrinsic endoplasmic pathway which is caspase-12-dependent. Deregulation of apoptosis has been implicated in various cardiovascular disease including ischemia-reperfusion, myocardial infarction, end-stage heart failure, and adriamycin cardiomyopathy.^7-10^

In patients who suffer from cardiac failure, the levels of NPs are found to be elevated, and high concentrations of NPs predict poor long-term survival.^11-14^ Evidence indicates that ANP and CNP inhibit vascular smooth muscle cell growth,^15-17^ and ANP, BNP and CNP induce apoptosis in cell cultures.^18-22^ Given that NPR3 interacts with all 3 natriuretic peptides, these results raise a hypothesis that NPR3 may be involved in apoptosis. In a recent study, we comprehensively re-sequenced NPR3 and found 7 novel non-synonymous single-nucleotide polymorphisms.^23^ In this study, we investigated the effects of NPR3 on the apoptosis of H9C2 cardiomyocytes and lineated a molecular mechanism of NPR3-mediated apoptosis involved.

## Materials and methods

### Cell lines and culture conditions

The rat embryonic ventricular myocardial cells (H9C2) and non-cardiomyocyte MCF7, T47D and Panc-1 cells were cultured in Dulbecco's modified Eagle's medium (DMEM) supplemented with 10% fetal bovine serum, 100U/ml penicillin and 100μg/ml streptomycin. Cells were grown in a humidified incubator containing 95% air and 5% CO_2_ at 37°C with media replenishment every 3 d.

### NPR3 knock-down and generation of stable cell line

H9C2 cells were seeded into a well of 6-well plate at 3 × 10^5^ in 2 ml. In the following day, the cells were transfected with sequence specific shRNA targeting NPR3 (OriGene, Cat. No. TR709485) by using FuGENE 6 transfection reagent (Roche, Cat. No. 1814443) according to the manufacturer's protocol. Following a 24 h transfection period, cells were selected with 10 ug/ml puromycin. After 10 d of puromycin selection, single colonies (well-isolated single clump of cells) were picked to generate stably transfected monoclonal cell lines.

### Western blot analysis

Whole cell lysates from NPR3 knock-down and control H9C2 cells treated with or without selective NPR3 agonist ring-deleted analog of atrial natriuretic factor (cANF) (Sigma, Cat. No. SCP0022) were subjected to electrophoresis on 7.5% Tris-Glycine extended gels. After electrophoresis, proteins were transferred to PVDF membranes (BioRad, Cat. No. 1620177), and the membranes were incubated with purified mouse anti-NPR3, Caspase-3, 8 and 9 (Cell Signaling,Cat. No. 9661, 9496 and 7237), breast cancer type 1 susceptibility protein (BRCA1) (Santa Cruz, Cat. No. sc-7867), cAMP response element-binding protein (CREB) (Cell Signaling, Cat. No. 9104), phospho-cAMP-dependent protein kinase (PKA) substrate (Cell Signaling, Cat. No. 9621) and tumor necrosis factor α (TNF-α) (Cell Signaling, Cat. No. 3707) antibodies, followed by incubation with a secondary antibody, and visualized by the ECL Western blotting detection system (Amersham).

### Immunofluorescent staining

Cells were plated on pre-coated glass coverslips in 12-well plates at 6 × 10^4^ per well for 24 h. Cells were exposed to 5 Gy of X-ray, fixed for 15 minutes in 4% paraformaldehyde and permeabilized for 10 minutes with 0.2% Triton X-100. The fixed cells were incubated for 1 hour in blocking solution (5% BSA with 0.1% triton X-100 in phosphate buffered saline). Immunostaining was performed by incubating the slides with anti-BRCA1 and phospho-Ser139-H2AX (Cell Signaling, Cat. No. 2577) at 4°C overnight, followed by secondary antibody for 1 hour at room temperature. Then, stained cells were mounted by mounting agent with DAPI.

### Assessment of cell viability by 3-(4,5-dimethylthiazol-2-yl)-5-(3-carboxymethoxyphenyl)-2- (4-sulfophenyl)-2H-tetrazolium, inner salt (MTS) assay

Before experimental intervention, confluent cultured cells were serum starved for 24 hours. The starved cells were then treated with serial concentration of H_2_O_2_ for 18 hours. Then, Cell Titer 96®Aqueous One Solution Reagent (Promega, Cat. No. G3580) was added to each well according to the manufacturer's instructions. After 3 hours incubation the cell viability was determined by measuring the absorbance at 490nm using a plate reader. Three independent survival experiments were performed for each experimental condition.

### Statistics

Data from quantitative experiments is presented as the mean ± SE. Statistical differences between the mean of cell viability studies of H9C2 cell groups were analyzed using unpaired Student's t-test (P < 0.05).

## Results

### Knockdown of NPR3 in H9C2 cells enhances the activities of caspase-3, -8 and -9

**Figure 1.**
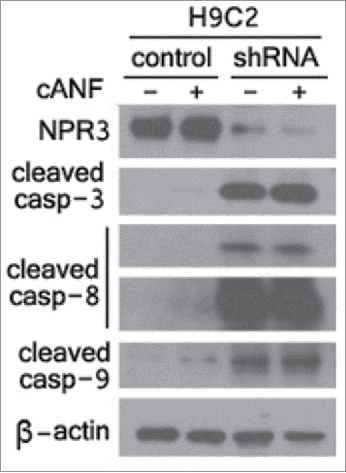
Enhancement of caspase activities in natriuretic peptide receptor 3 (NPR3) knock-down cardiomyocyte H9C2 cells. Immunoblot analysis of NPR3 and caspases was performed in lysates of NPR3 knock-down and control H9C2 cells treated with or without a selective NPR3 agonist ring-deleted analog of atrial natriuretic factor (cANF).

To determine whether NPR3 is involved in apoptosis, we first generated stable H9C2 cardiomyocyte cell lines expressing shRNA to knockdown NPR3. Knockdown efficiency was confirmed by western blot (Fig. 1). To determine whether NPR3 expression or treatment with a selective NPR3 agonist cANF affects apoptosis, caspase-3, caspase-8, and caspase-9 activities were examined. Compared with the controls, NPR3 knockdown significantly increased the activities of caspase-3, caspase-8, and caspase-9 (Fig.1), suggesting that NPR3 knockdown induces both the extrinsic and intrinsic apoptotic pathways. Unexpectedly, cANF treatment only weakly up-regulates activities of these caspase members, suggesting that the potential for inducing apoptosis is much higher in NPR3 knockdown than with ligand stimulation.

### NPR3 protects H9C2 cells from H_2_O_2_-induced cell death

**Figure 2.**
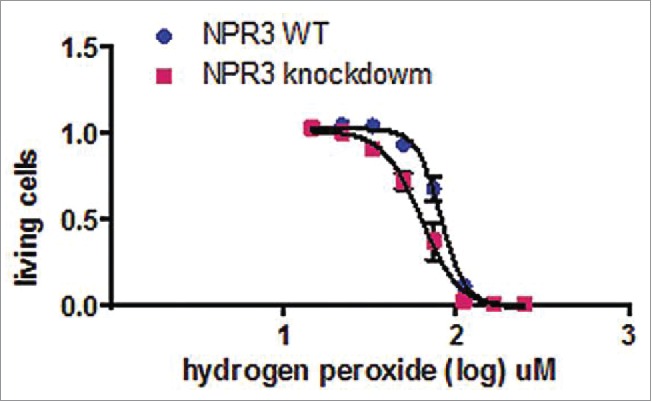
Effects of H_2_O_2_ on NPR3 knock-down H9C2 cell viability. Cells were incubated with increasing concentrations of H_2_O_2_ (0 to 250 nM) for 18 hours. Cell viability was measured by MTS assay. A significant decrease in cell viability with increased H_2_O_2_ concentration was observed in NPR3 knock-down H9C2 cells (P < 0 .05).

We examined the influence of NPR3 knockdown on the induction of apoptosis triggered by oxidative insult. H9C2 cardiomyocytes were treated with serial concentration of hydrogen peroxide (H_2_O_2_) for 18 h. Cell viability studies were performed using MTS assay. The results indicate that NPR3 knockdown was more sensitive to H_2_O_2_ than the controls (Fig. 2), suggesting that NPR3 protects H9C2 cells from apoptosis triggered by H_2_O_2_. These results are in agreement with the activity assay of caspase family members shown in Figure 1.

### BRCA1 and TNF-α are 2 main components of NPR3-dependent apoptotic pathway in H9C2 cells

#### Knockdown of NPR3 and treatment of cANF up-regulate expression levels of BRCA1

**Figure 3.**
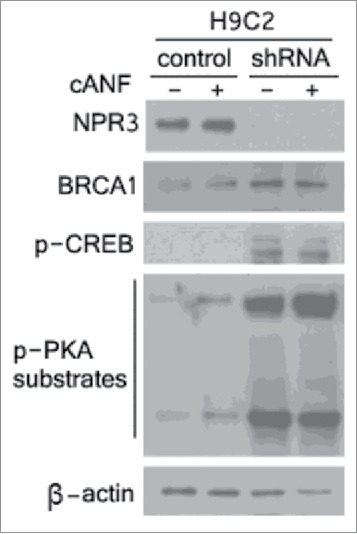
Upregulation of breast cancer type 1 susceptibility protein (BRCA1), cAMP response element-binding protein (CREB) and cAMP-dependent protein kinase (PKA) in NPR3 knock-down H9C2 cells. Immunoblot analysis of NPR3, BRCA1, CREB and PKA was performed in lysates of NPR3 knock-down and control H9C2 cells treated with or without a selective NPR3 agonist Canf.

Next, we studied the mechanism by which NPR3 protects cardiomyocytes from apoptosis. Evidence indicates that NPR3 is coupled to adenylyl cyclase though G_i_-protein and acts to decrease intracellular cAMP levels.^1^ Furthermore, studies have shown that there exists a cAMP-responsive element (CRE) in the BRCA1 proximal promoter between −176 and −169 base pairs that acts as a constitutive transcriptional element for BRCA1 expression.^24^ The critical role of CRE in BRCA1 promoter activity prompted us to examine whether NPR3 regulates BRCA1 expression though its effect on cAMP levels. Interestingly, when we analyzed the BRCA1 expression, knockdown of NPR3 significantly increased BRCA1 expression (Fig. 3). As reported before,^25^ we indirectly examined cAMP levels by measuring protein kinase A (PKA) activity. As shown in Figure 3, knockdown of NPR3 increases the phosphorylation of PKA substrates, suggesting that knockdown of NPR3 up-regulated cAMP levels. Furthermore, it has been reported that, in addition to CRE site on the promoter of BRCA1, cAMP responsive element-binding protein (CREB) is also important for BRCA1 expression.^26^ We analyzed CREB and found that knockdown of NPR3 dramatically increased CREB activity (Fig. 3). The effects of cANF treatment on both the activation of PKA substrates and activity of CREB were weak. Taken together, these results suggest that knockdown of NPR3 leads to up-regulation of BRCA1 through increasing cAMP levels and CREB activity.

#### BRCA1 mainly accumulates in cytoplasm, instead of nuclei in H9C2 cardiomyocytes

**Figure 4.**
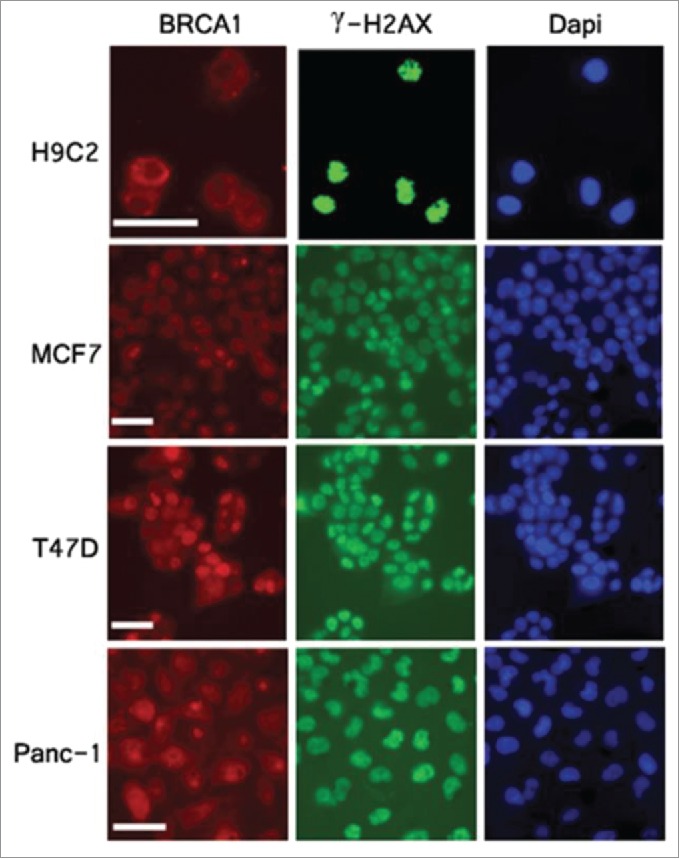
Cytoplasmic localization of BRCA1 in H9C2 cardiomyocytes. Cells were exposed to 5 Gy of X-ray and subjected to immunofluorescent analysis 1 hour after irradiation with anti- BRCA1 (red), γH2AX (green) antibodies. Nuclei were stained with DAPI (blue). Scale bar, 50 um.

Recent studies reveal that BRCA1 is a nuclear-cytoplasmic shuttling protein.^27-29^ In addition to the central role of double-strand break DNA repair, BRCA1 plays a role in apoptosis. Overexpression of BRCA1 induces apoptosis, a process that depends on its nuclear export.^28,29^ Furthermore, cytoplasmic BRCA1 accumulation alone induces apoptosis.^29^ BRCA1 functions can be controlled by discrete subcellular compartments. When in the nucleus, BRCA1 plays a role in DNA repair. In contrast, BRCA1 serves an apoptotic function when in cytoplasm. Given that knockdown of NPR3 up-regulates BRCA1 levels and promotes apoptosis, we sought to determine whether BRCA1 is cytosolic localization in H9C2 cardiomyocytes. Double immunofluorescence was performed in widely different cell lines, including H9C2 cardiomyocytes, MCF-7 and T47D human breast cancer cells, and BxPC-3 and PANC-1 pancreatic cancer cells to correlate BRCA1 subcellular localization with IR-induced γ-H2AX foci, a marker of double strand DNA breaks and nuclear localization, 1 hour after 5 Gy ionizing radiation. As shown in Figure 4, BRCA1 dispersed nuclear localization in MCF-7, T47D, BxPC-3 and PANC-1 cells. In contrast, BRCA1 exhibited predominantly cytoplasmic localization in H9C2 cardiomyocytes, highlighting a crucial difference in BRCA1 localization in H9C2 cells

.

#### Knockdown of NPR3 / cANF treatment / high expression level of BRCA1 promote upregulation of TNF-α

**Figure 5.**
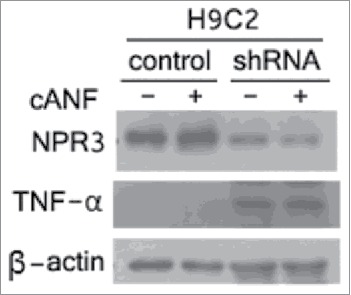
Up-regulation of tumor necrosis factor α (TNF-α) in NPR3 knock-down H9C2 cells. Immunoblot analysis of NPR3 and TNF-α was performed in lysates of NPR3 knock-down and control H9C2 cells treated with or without a selective NPR3 agonist cANF.

It has been shown that the presence of germline mutations in BRCA1 was associated with a selective deficiency in TNF-α production.^30^ Furthermore, recent studies indicate TNF-α provokes cardiomyocyte apoptosis through activation of both the extrinsic and intrinsic apoptotic pathways with resultant activation of different members of the caspase family.^31^ Thus, an alternative hypothesis is that up-regualtion of BRCA1 resulting from knockdown of NPR3 may lead to an increase in protein expression of TNF-α which triggers both of extrinsic and intrinsic apoptotic pathways in H9C2 cardiomyocytes. To test this hypothesis, we analyzed the expression of TNF-α. As shown in Figure 5, compared with the controls, TNF-α was significantly up-regualted in NPR3 knockdown cells. Thus, the up-regulation of activities of caspase-3, 8, and 9 (Fig. 1) may be a consequence of elevated TNF-α expression.

## Discussion

Upregulation of NP system including NPs and their receptors is a hallmark of many cardiovascular diseases including heart failure.^32^ Apoptosis is widely recognized as an important factor contributing to heart failure.^33^ However, the precise mechanism by which natriuretic peptide receptors exert their actions to apoptosis remains to be elucidated. Here we report an elegant mechanism by which NPR3 exerts its effects on known steps of the apoptotic process in H9C2 cardiomyocytes, thus defining a link between NPR3 and the execution of apoptosis. The first important finding of this study is the up-regulation of the activities of caspase family members including caspase-3, 8, and 9 in NPR3 knockdown H9C2 cardiomyocytes compared with the controls. Once these 3 caspase-3, 8, and 9 are activated, the execution phase of apoptosis is triggered. These results are in agreement with the cytotoxicity assay shown in Figure 2. To study the involvement of NPR3 in regulating apoptosis, we analyzed downstream targets leading to apoptosis. Given that NPR3 inhibits adenylate cyclase and down-regulates cAMP levels, and both the CRE site and CREB are important for the constitutive expression of BRCA1, we analyzed BRCA1 expression. Here, we show for the first time that knockdown of NPR3 dramatically increases BRCA1 expression in H9C2 cardiomyocytes. Although the biological significance of the upregulation of BRCA1 in H9C2 cardiomyocytes is unknown, it is well established that BRCA1 is a nuclear-cytoplasmic shuttling protein, and controls DNA repair when nuclear and regulates apoptotic process when cytoplasmic. Results in the present study show for the first time that H9C2 cardiomyocytes exhibit predominantly cytoplasmic BRCA1 (Fig. 4), which enables them to undergo BRCA1-mediated apoptosis. In contrast, non-myocardial BxPC-3 and PANC-1 pancreatic cancer cells and MCF-7 and T47D human breast cancer cells exhibit pronounced nuclear BRCA1. It has been shown that germline mutations of BRCA1 are associated with deficient TNF-α production. Here, we show that up-regulation of BRCA1 resulting from NPR3 knockdown in H9C2 cardiomyocytes significantly increases TNF-α expression. Studies have shown that TNF-α provokes cardiomyocyte apoptosis through activation of both the extrinsic and intrinsic apoptotic pathway. Indeed, the results of this study indicate that knockdown of NPR3 with resultant elevated levels of TNF-α in H9C2 cardiomyocytes significantly activates both the extrinsic and intrinsic apoptotic pathways through upregulation of the activities of caspase-3, 8, and 9 shown in Figure 1. Thus, cytotoxic signal transduction by NPR3 knockdown in H9C2 cardiomyocytes proceeds through the following 4 steps in order: 1) up-regulation of cAMP levels; 2) increasing levels of cytoplasmic BRCA1 by elevated CREB activity; 3) increasing levels of TNF-α; 4) activation of both the extrinsic and intrinsic apoptotic pathways through activated caspase-3, 8, and 9 triggered by TNF-α.

The effects of cANF on inhibition of adenylate cyclase activity have been studied in rat aorta, brain striatum, anterior pituitary and adrenal cortical membrane preparations in a concentration-dependent manner.^34^ The maximal inhibition was about 50–60%. It is now accepted that cANF is a specific and selective agonist of NPR3. In this study, we note that the apoptosis-inducing capacity of NPR3 knockdown in H9C2 cells is much higher than controls stimulated with cANF (Fig. 1), suggesting the effects of NPR3 expression levels on apoptosis are much more pronounced than that of ligand stimulation. Therefore, NPR3 mutation carrier may be at a previously unrecognized risk of heart failure.

In addition to clarifying the role of NPR3 in apoptosis, the results of this study provide several new insights into the mechanism by which it acts. In particular, we have observed BRCA1 cytoplasmic localization in H9C2 cardiomyocytes, which distinguishes them from non-cardiomyocytes. As reported previously, BRCA1 cytoplamic accumulation in breast cancer cells plays an important role in apoptosis. BRCA1 cytoplamic localization in H9C2 cardiomyocytes raises a hypothesis that cardiomyocytes may be more sensitive to DNA-damaging agents than non-cardiomyocytes. This is consistent with clinical reports indicating that the risk of death from heart disease in patients treated with radiation therapy is increased from 1.5 to 3.0 times that of un-irradiated patients.^35^ Breast cancer radiotherapy increases the risk of developing ischemic heart disease.^36,37^

In this study, we show for the first time that NPR3, one of the NP system components, is involved in apoptosis through BRCA1 and TNF-α. Apoptosis is an important mechanism of cell death in heart failure. However, the underlying mechanisms by which the heart loses cardiomyocytes in heart failure remain elusive. The results in this study shed light on the role of NPR3 in the execution of apoptosis, and establish the first molecular link between NP system and programmed cell death.

## Abbreviations

*BRCA1*: breast cancer type 1 susceptibility protein
*cAMP*: Cyclic adenosine monophosphate
*NPR3*: natriuretic peptide receptor-3
*NPs*: natriuretic peptides
*TNF-α*: tumor necrosis factor α
*PKA*: protein kinase A
*CRE*: cAMP responsive element
*CREB*: cAMP responsive element-binding protein
